# Fatty acid-binding protein 5 (FABP5) modulates limbal epithelial cell homeostasis by regulating the expression of key genes under both normal and inflammatory conditions*, in vitro*

**DOI:** 10.1371/journal.pone.0347228

**Published:** 2026-04-28

**Authors:** Maryam Amini, Shao-Lun Hsu, Tanja Stachon, Zhen Li, Ning Chai, Fabian N. Fries, Berthold Seitz, Swarnali Kundu, Shweta Suiwal, Nóra Szentmáry

**Affiliations:** 1 Dr. Rolf M. Schwiete Center for Limbal Stem Cell and Congenital Aniridia Research, Saarland University, Homburg/Saar, Germany; 2 Department of Experimental Ophthalmology, Homburg/Saar, Germany; 3 Department of Ophthalmology, Saarland University Medical Center, Homburg/Saar, Germany; Cedars-Sinai Medical Center, UNITED STATES OF AMERICA

## Abstract

Congenital aniridia, a rare disorder caused by PAX6 haploinsufficiency, is characterized by progressive, vision-threatening aniridia-associated keratopathy (AAK) with limbal epithelial dysfunction and chronic inflammation. Downregulation of fatty acid binding protein 5 (FABP5) has been reported in conjunctival cells of congenital aniridia patients and in limbal epithelial cells (LECs) of the PAX6 siRNA knockdown model. We aimed to investigate the effects of FABP5 deficiency on LECs gene expression, without or with inflammatory stimuli. To achieve FABP5 knockdown, human primary LECs were transfected with FABP5 siRNA, using Lipofectamine 2000. Inflammation was induced 48 hours after transfection by incubating cells with 2 µg/mL lipopolysaccharides (LPS) or 1 ng/ml IL-1β. Thereafter, gene and protein levels were examined using qPCR, Western blot and ELISA. Significant downregulation of *PAX6*, *KRT3* and *MMP2* and upregulation of *KRT12* mRNA level was observed upon FABP5 knockdown (*p* ≤ 0.022). Under inflammatory conditions (IL-1β or LPS treatment), FABP5 knockdown led to reduced *PAX6, FOSL2, IL-6, PTGES2, KRT3, MAPK3* and *MMP2* (*p* ≤ 0.048) and increased *VEGFα* and *CRABP2* mRNA expression levels compared to control LECs (*p* ≤ 0.034). Following FABP5 knockdown, reduced PAX6, IL-6 and KRT3 protein levels were confirmed in absence of inflammatory stimuli and in cells treated with IL-1β (*p* ≤ 0.034). Our results suggest a novel role of FABP5 protein in AAK progression by controlling expression levels of genes and proteins involved in LEC differentiation. In addition, under inflammatory conditions, FABP5 deficiency affects key transcription factors (*PAX6, FOSL2*), genes regulating LEC migration, differentiation and cell maintenance (*KRT3, VEGFα, MAPK3, CRABP2*), and genes involved in inflammation (*IL-6, PTGES2*).

## Introduction

Fatty acid binding proteins (FABPs) are intracellular lipid binding proteins, that play a crucial role in transporting fatty acids between cellular compartments [1]. Transferring lipids and their derivatives to the nucleus by FABPs facilitates the expression of a wide variety of genes [2]. The epidermal member of FABP family, commonly referred to as FABP5, C-FABP, E-FABP or PA-FABP, is expressed in a broad range of tissues including skin, brain, lens, ocular surface, limbal epithelial cells, retina, bone, liver, lungs, spleen, kidneys, tongue, adipose tissues, cardiac and intestinal tissues and breast [3]. FABP5 has been known to interact indirectly with ion channels and receptors on the membrane, enzymes and genes by modulating the fatty acid concentrations [4]. The role of this protein has been reported in several diseases such as psoriasis [5], Alzheimer‘s disease [6], cancers (prostate and breast cancer) [7] and metabolic disorders (obesity and insulin resistance) [8].

FABP5 is known to be involved in cell proliferation and differentiation by controlling the expression of several genes. The role of FABP5 in regulating cell cycle has been shown by the work of Yu and his colleagues [9]. Additionally, the FABP5 protein is involved in gene transcription by activating PPARs, which further influence gene expression related to lipid metabolism, cellular differentiation and proliferation [10]. Furthermore, FABP5 overexpression was shown to be linked with enhanced proliferation, survival and migration of cancer cells [11]. In addition, the contribution of FABP5 in inflammation and cytokine production has been investigated [12–14]. Several studies reported its role in mediating inflammatory responses by modulating PPARγ [10,15].

Congenital aniridia is a rare genetic disorder, mostly caused by PAX6 haploinsufficiency and characterized by iris hypoplasia. This panocular disease is manifested by photophobia, nystagmus, aniridia associated keratopathy (AAK), secondary glaucoma, juvenile cataract, macular and optic nerve head hypoplasia, and visual impairment [16]. In previous studies, FABP5 downregulation has been reported in conjunctival cells of congenital aniridia patients [17], as well as in the PAX6 siRNA knockdown model of limbal epithelial cells (LECs) [18]. AAK is marked by progressive dysfunction of the limbal epithelial compartment accompanied by a chronic inflammatory microenvironment [19]. Moreover, a significant upregulation of inflammatory cytokines, such as interleukin (IL-) 1β, IL-9, IL-17A; eotaxin; basic fibroblast growth factor (bFGF/FGF2); and macrophage inflammatory protein 1α (MIP-1α/CCL3) have been detected in tear fluid of congenital aniridia patients [20].

Inducing inflammation in cell cultures using lipopolysaccharides (LPS) or interleukin (IL)-1β, is well established and commonly used in many studies. LPS induces signaling through TLR4, which initiates inflammatory responses as explained by Page et al. [21]. Binding of IL-1β to its receptor, IL-1R1, also initiates the inflammatory response in a similar signaling pathway as TLR4 activation [22,23].

The purpose of this study was to investigate whether FABP5, a downstream protein of PAX6, acts as a mediator of well-established aniridia-associated alterations in gene and protein expression. To address this, we performed FABP5 knockdown in healthy limbal epithelial cells and analyzed the expression of key genes involved in epithelial maintenance, differentiation, and proliferation under both inflammatory and non-inflammatory conditions.

## Materials and methods

### Primary limbal epithelial cell isolation and culture

The research project received a favorable vote from the Saarland Ethics Commission (162/23). All experiments were conducted between 01.12.2022 and 17.03.2025. The samples were collected from 01.12.2022 to 30.11.2024. Primary limbal epithelial cells (LECs) were isolated from corneoscleral rims obtained from the Klaus Faber Center for Corneal Diseases, including the Lions Eye Bank, after their use in corneal transplantation procedures. In short, a 1.5 mm punch (Kai Medical, Solingen, Germany) was used to excise tissue from the limbal region and the tissue fragments were incubated overnight with 0.5 mg/ml Collagenase A (Roche Pharma AG, Basel, Switzerland), at 37 ºC, 5% CO_2_. The following day, the cell suspension was passed through a 20 µm CellTrics® filter (Sysmex Partec GmbH, Görlitz, Germany). Cells attached to the filter were washed with 3 ml of trypsin-EDTA solution (Sigma-Aldrich GmbH, Deisenheim, Germany) and incubated for 10 minutes at 37 °C and 5% CO₂. Trypsin was deactivated by adding Dulbecco’s Modified Eagle Medium (DMEM) (Cat. Nr. 11594426, ThermoFischer scientific, Inc.), containing 10% FCS. After centrifugation at 200g for 5 minutes, cells were cultured in Keratinocyte growth medium (KSFM) (Cat. Nr. C-20111, Promocell, Heidelberg, Germany), at 37 ºC, 5% CO_2_. The age and gender of the donors in our study is displayed in **Table 1**. The human tissue experiments were conducted in accordance with the ARVO Best Practices for Using Human Eye Tissue in Research (November 2021).

**Table 1:** Age and gender of the donors in the study.

	Age (years)	Gender
Donor 1	90	Female
Donor 2	n/a	Female
Donor 3	n/a	Female
Donor 4	89	Male
Donor 5	71	Female
Donor 6	88	Female
Donor 7	n/a	Male
Donor 8	65	Female

n/a: not available

### Primary LEC transfection and induction of inflammation

**Fig 1:**
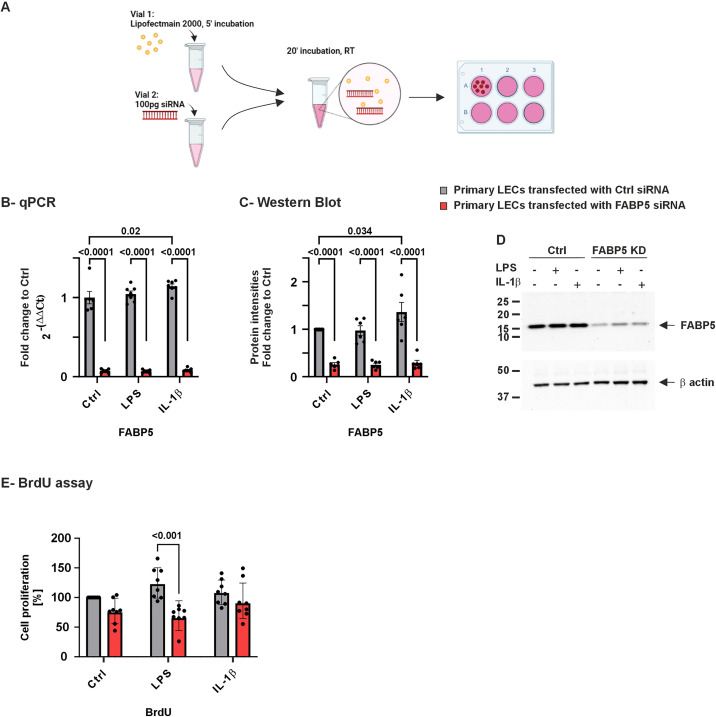
FABP5 mRNA and protein expression in primary human LECs 72h after FABP5 siRNA ± LPS/IL-1β. Schematic view of LECs transfection protocol **(A)**, (min: minutes; RT: Room temperature). FABP5 mRNA expression (B) and protein level (C) in LECs transfected with FABP5 siRNA or control (ctrl) siRNA. Western blot image from a single biological replicate **(D)**. (E) shows the results of the proliferation assay in LECs transfected with either FABP5 siRNA or control siRNA. Data are shown as mean ± SEM. Statistical analysis has been performed using two-way ANOVA followed by Tukey test (*n* *= 7*). *p*-values below 0.05 were considered statistically significant. FABP5 expression was significantly lower at mRNA and protein levels following FABP5 siRNA knockdown, without or with LPS or IL-1β treatment, compared to ctrl siRNA LECs cultures (*p* < 0.0001). In addition, IL-1β treatment significantly increased FABP5 mRNA and protein levels in the ctrl siRNA LECs cultures (*p* ≤ 0.034).

LECs were transfected using Lipofectamine 2000 transfection reagent (Cat. Nr. 11668027, Invitrogen; ThermoFisher Scientific, Inc.), following the manufacturer’s instructions. To obtain the best transfection efficiency, after reaching 70–80% confluency, cells were transfected using 100 pg/ml of either control (ctrl) siRNA (Cat. Nr. 4390843, ThermoFisher Scientific, Inc.) or FABP5 siRNA (sequence of sense strand: GGAAUAGCUUUGCGAAAAAtt and anti-sense strand: UUUUUCGCAAAGCUAUUCCca, Cat. Nr. 4390824, ThermoFischer scientific, Inc.). Transfected cells were then incubated at 37ºC, and 5% CO_2_ for 24 hours (**Fig 1A**). The day after the medium was changed and cells were collected 72 hours after transfection for further investigations.

To assess the impact of FABP5 knockdown on gene expression under inflammatory conditions, the culture medium was replaced 48 hours after transfection with KSFM supplemented with either 2 µg/mL lipopolysaccharide (LPS; Sigma-Aldrich, St. Louis, USA) or 1 ng/ml animal-free recombinant human IL-1β (PeproTech, Inc., Rosemont, USA) for 24 hours. Finally, cells were collected for further analysis 72 hours after transfection, identical to the cultures without inflammatory stimuli.

### BrdU assay

To assess the impact of various conditions on cell proliferation, a colorimetric BrdU Cell Proliferation ELISA kit (Merck KGaA, Darmstadt, Germany) was employed in accordance with the manufacturer’s instructions. Absorbance was measured using the Tecan Infinite F50 Absorbance Microplate Reader (Tecan Group AG, Männedorf, Switzerland), the data were normalized to the group of ctrl siRNA-treated cells, which will be referred to as “ctrl siRNA LECs”.

### Protein extraction, RNA extraction and reverse transcription

For RNA and total protein isolation, DNA/RNA/Protein Purification Plus Micro Kit (Cat. Nr. 47700, Norgen Biotek CORP. Canada) was used. All steps were performed following the manufacturer’s protocol. RNA concentrations were measured by absorbance at 260 nm using a UV/VIS spectrophotometer (Analytik Jena AG, Jena, Germany). RNA and protein samples were stored at −80°C until further experiments. Reverse transcription was performed using 1 μg RNA and One Taq® RT-PCR Kit (New England Biolabs INC, Frankfurt, Germany). cDNA samples were stored at −20°C.

### Quantitative PCR (qPCR)

Quantitative PCR (qPCR) experiments were performed using the SYBR Green kit (Qiagen N.V., Venlo, Netherlands). For each reaction, a mixture of 9 µl reaction buffer, containing 1 µl of the specific primer solution (QuantiTect Primer Assay, Qiagen N.V., Venlo, Netherlands), 5 µl SYBR Green Mix and 3 µl nuclease-free water was prepared and set up in a 96 well qPCR plate. Furthermore, 1 µl cDNA template was added to the corresponding well. Plates were then placed in a QuantStudio 5 real-time PCR system (Thermo Fisher Scientific, Waltham, MA, USA). The amplification conditions were as follows, 95°C for 10s, 60°C for 30s, and 95°C for 15s (40 cycles). Results were represented and analyzed as fold change, compared to the ctrl siRNA treated cells (2^- (Δ ΔCt)^). Primers used in this study are listed in Table 2.

**Table 2:** Primers used for qPCR.

Primer	Gene Globe ID	Manufacturer
Hs_FABP5_1_SG QuantiTect Primer Assay	QT0022556	Qiagen N.V., Venlo, Netherlands
Hs_VEGFA_1_SG QuantiTect Primer Assay	QT01010184	Qiagen N.V., Venlo, Netherlands
Hs_MAPK1_1_SG QuantiTect Primer Assay	QT00065933	Qiagen N.V., Venlo, Netherlands
Hs_MAPK3_2_SG QuantiTect Primer Assay	QT02589314	Qiagen N.V., Venlo, Netherlands
Hs_AKT1_ 1_SG QuantiTect Primer Assay	QT00085379	Qiagen N.V., Venlo, Netherlands
Hs_MMP2_vb.1_SG QuantiTect Primer Assay	QT02395778	Qiagen N.V., Venlo, Netherlands
Hs_MMP9_1_SG QuantiTect Primer Assay	QT00040040	Qiagen N.V., Venlo, Netherlands
Hs_MKI67_1_SG QuantiTect Primer Assay	QT00014203	Qiagen N.V., Venlo, Netherlands
Hs_KRT3_1_SG QuantiTect Primer Assay	QT00050365	Qiagen N.V., Venlo, Netherlands
Hs_KRT12_1_SG QuantiTect Primer Assay	QT00011949	Qiagen N.V., Venlo, Netherlands
Hs_CRABP2_1_SG QuantiTect Primer Assay	QT00063434	Qiagen N.V., Venlo, Netherlands
Hs_ABCG2_1_SG QuantiTect Primer Assay	QT00073206	Qiagen N.V., Venlo, Netherlands
Hs_PAX6_1_SG QuantiTect Primer Assay	QT00071169	Qiagen N.V., Venlo, Netherlands
Hs_TP53_1_SG QuantiTect Primer Assay	QT00060235	Qiagen N.V., Venlo, Netherlands
Hs_TP63_2_SG QuantiTect Primer Assay	QT02424051	Qiagen N.V., Venlo, Netherlands
Hs_FOSL2_1_SG QuantiTect Primer Assay	QT01000881	Qiagen N.V., Venlo, Netherlands
Hs_FOXC1_1_SG QuantiTect Primer Assay	QT00217161	Qiagen N.V., Venlo, Netherlands
Hs_PPARG_1_SG QuantiTect Primer Assay	QT00029841	Qiagen N.V., Venlo, Netherlands
Hs_RELA_2_SG QuantiTect Primer Assay	QT02324308	Qiagen N.V., Venlo, Netherlands
Hs_IL1A_1_SG QuantiTect Primer Assay	QT00001127	Qiagen N.V., Venlo, Netherlands
Hs_IL1B_1_SG QuantiTect Primer Assay	QT00021385	Qiagen N.V., Venlo, Netherlands
Hs_IL16_1_SG QuantiTect Primer Assay	QT00075138	Qiagen N.V., Venlo, Netherlands
Hs_PTGES2_1_SG QuantiTect Primer Assay	QT00082068	Qiagen N.V., Venlo, Netherlands
Hs_TBP_1_SG QuantiTect Primer Assay	QT00000721	Qiagen N.V., Venlo, Netherlands

### Western blot analysis

Changes in intracellular protein concentrations were investigated using gel electrophoresis. Total protein concentration was measured by Bradford assay (Sigma-Aldrich, Merck KGaA, Darmstadt, Germany) and bovine serum albumin (BSA) was used as standard. The absorbances were measured at 595 nm wavelength using the Tecan Infinite F50 Absorbance Microplate Reader (Tecan Group AG, Männedorf, Switzerland) and protein concentrations were estimated using the linear equation of the standard curve obtained from BSA standard concentrations.

For electrophoresis, 4x Laemmli buffer was added to 20 µg protein and denaturation was performed by incubating samples at 95°C for 5 minutes. Samples were then loaded to NuPAGE ™ bis-tris precast 4–12% ready to use gel (ThermoFisher Scientific™ GmbH, Dreieich, Germany). Following protein separation, Trans Blot Turbo Transfer System (BioRad, Hercules CA, USA) was used to transfer the proteins onto a nitrocellulose membrane (BioRad, Hercules CA, USA). Membranes were incubated with primary antibodies diluted in WesternFroxx anti-rabbit or anti-mouse horseradish peroxidase (HRP) solution (BioFroxx GmbH, Einhausen, Germany), containing blocking reagent and secondary antibody (BioFroxx GmbH, Einhausen, Germany) overnight, at 4°C. On the next day, blots were washed 3 times with WesternFoxx washing solution (BioFroxx GmbH, Einhausen, Germany) and protein bands were visualized by incubation with Enhanced chemiluminescence (ECL buffers) (PerkinElmer, USA), using an iBright imaging system (Thermo Fisher Scientific, Darmstadt, Germany). The intensity of protein signals was analyzed by the iBright software. To ensure equal protein loading, the intensity of each protein band was normalized to the β-actin signal in the corresponding lane. Table 3 summarizes the Primary antibodies used in this study.

**Table 3:** Primary antibodies used for Western blot.

Antibody	Class	Dilution	Cat. No.	Manufacturer
FABP5	Polyclonal	1:1000	12348-1-AP	Proteintech, Martinsried, Germany
VEGFα	Polyclonal	1:1000	19003-1-AP	Proteintech, Martinsried, Germany
p44/42 MAPK (Erk1/2)	Monoclonal	1:1000	137F5	Cell signaling technology, Inc., Danvers, Massachusetts, USA
MMP2	Monoclonal	1:250	66366-1-Ig	Proteintech, Martinsried, Germany
Keratin K3/K76	Monoclonal	1:500	CBL218	MERCK, Darmstadt, Germany
Cytokeratin 12 (E-8)	Monoclonal	1:2000	sc-515882	Santa Cruz biotechnology, Inc.
PAX6	Polyclonal	1:1000	AB2237	Millipore, Watford, UK
FOSL2	Polyclonal	1:500	15832-1-AP	Proteintech, Martinsried, Germany
PTGES2	Polyclonal	1:500	10881-1-AP	Proteintech, Martinsried, Germany
β-actin	Polyclonal	1:10000	Ab8227	Abcam, Cambridge, UK

### ELISA

To examine the intracellular protein concentration of IL-6, Enzyme-linked Immunosorbent Assay (ELISA) was performed following cell lysis. First, Bovine IL-6 DuoSet ELISA kit (Cat. No. DY008, R&D, Minneapolis, USA) was used, following the manufacturer’s protocol. Then, 50 μg of total protein from each sample was added to a 96 well in duplicate. Protein concentrations were estimated using the IL-6 standard curve.

### Statistical analysis

Statistical analyses were performed using GraphPad Prism. Data are presented as mean ± SEM. All values were normalized within each experiment to ctrl siRNA-transfected cells cultured in KSFM without LPS or IL-1β, which were defined as the control group (ctrl siRNA LECs).

Outliers were assessed at the donor level using Grubbs’ test (α = 0.05), provided that the minimum sample size requirement for the test was met. Cells derived from each donor were cultured in separate wells and exposed to different experimental conditions. Accordingly, data were analyzed using a two-way ANOVA with siRNA treatment (ctrl siRNA vs. FABP5 siRNA) and inflammatory condition (KSFM, KSFM + LPS, KSFM + IL-1β) as within-subject factors. A full factorial model including main effects and the siRNA × condition interaction was applied. Post hoc comparisons were performed using Tukey’s multiple comparisons test with two-sided testing. The effects of FABP5 knockdown were assessed by comparing ctrl siRNA and FABP5 siRNA within each culture condition, while the impact of inflammatory stimulation was evaluated by comparing KSFM with the corresponding transfection group exposed to LPS or IL-1β. *p*-values below 0.05 were considered statistically significant.

## Results

### FABP5 knockdown efficiency in LECs and proliferation across different treatment groups

We first confirmed the significant downregulation of FABP5 mRNA and protein levels upon siRNA transfection by qPCR and western blot, in absence or presence of inflammatory stimuli (*p* < 0.0001 for both). LECs transfected with FABP5 siRNA showed ~ 90% reduction in *FABP5* mRNA expression and ~ 70% downregulation in protein levels in comparison to ctrl siRNA LECs. Besides, IL-1β treatment significantly increased *FABP5* mRNA (*p* = 0.02) and protein (*p* = 0.034) levels in the control LECs (Fig 1B-1D). The BrdU assay revealed a significant reduction in proliferation in FABP5 knockdown cells following LPS treatment (*p* < 0.001), while no significant changes were observed in any other treatment groups (Fig 1E).

### Factors with potential effect on epithelial cell proliferation and migration are affected following FABP5 knockdown

**Fig 2:**
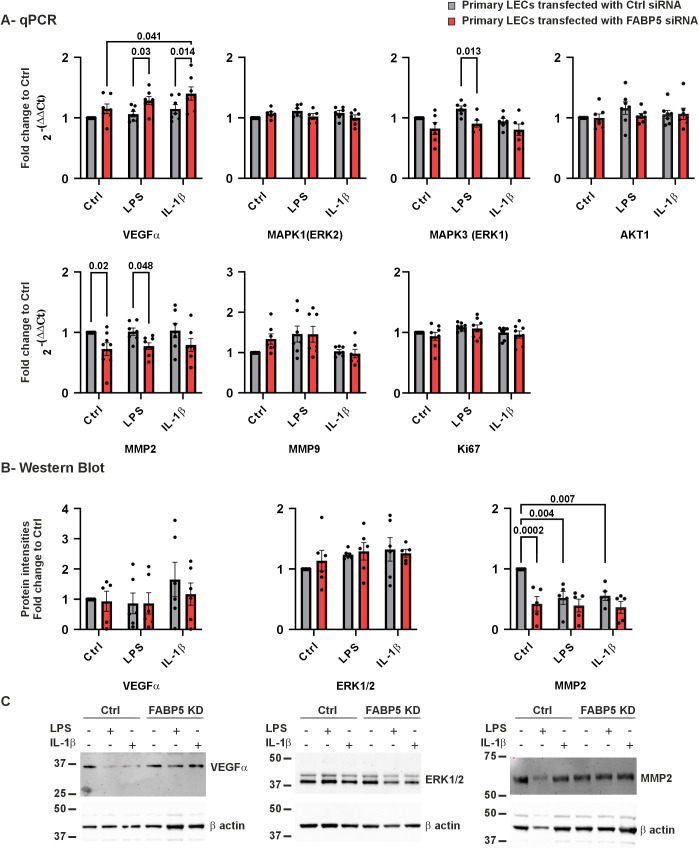
*VEGFα*, *MAPK1/3, AKT1, MMP2/9, Ki67 mRNA* and VEGFα, ERK1/2, MMP2 proteins in LECs after FABP5 siRNA ± LPS/IL-1β. *VEGFα, MAPK1, MAPK3, AKT1, MMP2* and *MMP9* mRNA expression (A) and VEGFα, ERK1/2 and MMP2 protein levels (B) in LECs transfected with FABP5 siRNA or control (ctrl) siRNA. Western blot image from a single biological replicate **(C)**. Data are shown as mean ± SEM. Statistical analysis has been performed using two-way ANOVA followed by Tukey test (*n = 7*). *p-*values below 0.05 were considered statistically significant. *VEGFα* mRNA expression was significantly higher in FABP5 knockdown LECs following LPS and IL-1β treatment, than in ctrl siRNA LECs after LPS and IL-1β treatment (*p =* 0.03; *p* = 0.014). *VEGFα* mRNA expression was significantly higher in FABP5 knockdown LECs following IL-1β treatment, than without treatment (*p* = 0.041). After LPS treatment, *MAPK3* mRNA expression was significantly lower in FABP5 knockdown LECs than in ctrl siRNA LECs (*p* = 0.013). *MMP2* mRNA expression was significantly lower in FABP5 knockdown LECs without treatment and following LPS treatment, than in ctrl siRNA LECs without treatment and after LPS treatment (*p* = 0.02; *p* = 0.048). MMP2 protein levels were significantly lower in FABP5 knockdown LECs without treatment, than in ctrl siRNA LECs without treatment (*p* = 0.0002). MMP2 protein levels were also significantly decreased after LPS and IL-1β treatment in ctrl siRNA LECs (*p* = 0.004 and 0.007).

An important characteristic of LECs is their ability to proliferate and migrate in order to generate the corneal epithelial cell layer and to modulate wound healing [24]. Several proteins are known to mediate proliferation and migration of epithelial cells including VEGFα, MAPK1, MAPK3, MMP2 and MMP9 [25–28]. As shown in Fig 2A, FABP5 knockdown LECs showed a significant increase in *VEGFα* mRNA expression under both inflammatory conditions compared to ctrl siRNA LECs (*p* = 0.03 and *p* = 0.014, respectively). Furthermore, the expression of *VEGFα* mRNA in FABP5 knockdown cells in IL-1β containing medium revealed a significant increase in *VEGFα* mRNA, compared to KSFM medium (*p* = 0.041).

In addition, LPS treated FABP5 knockdown cells showed significantly lower expression levels of *MAPK3* mRNA compared to ctrl siRNA LECs with LPS treatment (*p* = 0.013). FABP5 deficiency also resulted in lower expression levels of *MMP2* mRNA using KSFM medium and upon LPS-induced inflammation, compared to ctrl siRNA LECs (*p* = 0.0002; *p* = 0.048). *MAPK1* and *AKT1* expressions were not significantly altered in any of the comparisons (*p* ≥ 0.099). In addition, mRNA level of the proliferation marker *Ki67* also did not differ significantly between any of the groups (*p* ≥ 0.124) (Fig 2A).

In contrast to gene expression analysis, we were not able to detect any significant alterations in VEGFα and ERK1/2 protein levels (Fig 2B-2C). Without inflammatory stimuli, MMP2 protein level was significantly lower in FABP5 knockdown LECS, than in ctrl siRNA LECs (*p* = 0.002). In ctrl siRNA LECs, presence of LPS or IL-1β in the medium also resulted in a lower MMP2 protein level, than KSFM medium (*p* = 0.004 and 0.007 respectively). However, under LPS-induced inflammatory conditions, reduced MMP2 protein expression could not be confirmed in FABP5 knockdown LECs compared with ctrl siRNA LECs (Fig 2B-2C).

### FABP5 is controlling genes with an effect on LEC differentiation and cell maintenance

**Fig 3:**
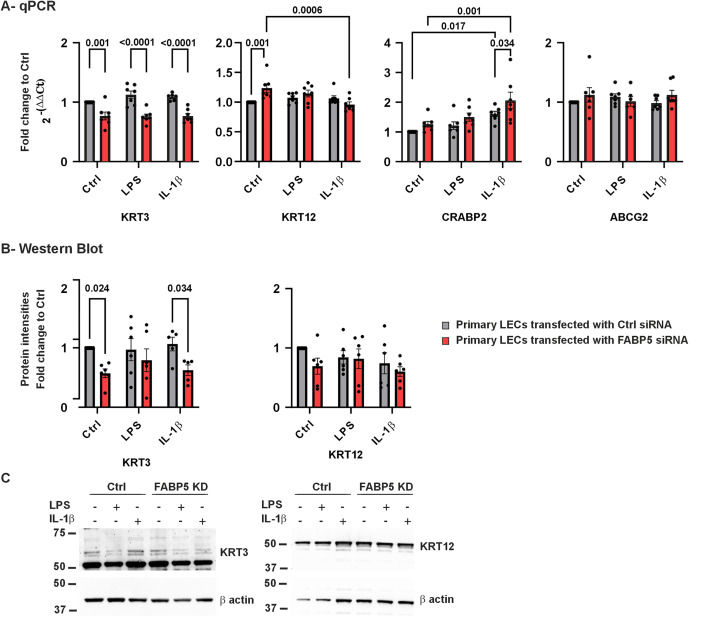
*KRT3, KRT12, CRABP2, ABCG2* mRNA (A) and KRT3/12 proteins (B) in LECs with FABP5/control siRNA. Western blot image from a single biological replicate **(C)**. Data are shown as mean ± SEM. Statistical analysis has been performed using two-way ANOVA followed by Tukey test (*n = 7*). *p*-values below 0.05 were considered statistically significant. *KRT3* mRNA expression was significantly lower following FABP5 siRNA knockdown, without or with LPS or IL-1β treatment, compared to ctrl siRNA LECs cultures (*p* < 0.001 for all). Nevertheless, *KRT12* mRNA level was significantly higher in FABP5 knockdown LECs, than in control siRNA transfected LECs, without treatment (*p* = 0.001). IL-1β treatment significantly increased *KRT12* mRNA expression in FABP5 knockdown LECs (*p* = 0.0006). *CRABP2* mRNA expression was significantly higher both in ctrl siRNA LECs and in FABP5 knockdown LECs following IL-1β treatment, than without treatment (*p* = 0.017; *p* = 0.001). Furthermore, following IL-1β treatment, *CRABP2* mRNA expression was significantly higher in FABP5 knockdown LECs than in ctrl siRNA LECs (*p* = 0.034). CRABP2 protein could not be detected in any of the analyzed groups, as no band was observed upon blot incubation; however, the CRABP5 protein band was clearly visible in Michigan Cancer Foundation-7 (MCF-7) cells, which are known to express CRABP2 at significant levels and were therefore chosen as the positive control. (**Supplement 1**). Without treatment or with IL-1β treatment, KRT3 protein level was significantly lower following FABP5 siRNA knockdown, than in ctrl siRNA LECs cultures (*p* ≤ 0.034).

We also studied the effects of FABP5 deficiency on mRNA expression levels of genes participating in epithelial cell maintenance, such as *KRT3, KRT12, CRABP2* and *ABCG2* (Fig 3). *KRT3*, which is known as a differentiation marker of LECs, showed significantly reduced mRNA expression level in FABP5 deficient cells using KSFM medium, and in medium containing LPS or IL-1β (*p* < 0.001 for all). KRT3 protein level was also downregulated upon FABP5 knockdown in normal and in IL-1β containing medium (*p* = 0.024; *p* = 0.034) (Fig 3A-3C).

A significant upregulation of *KRT12* mRNA was observed in FABP5 deficient cells in KSFM medium (*p* = 0.001). IL-β treatment led to a downregulation of *KRT12* mRNA in FABP5 deficient cells (*p* = 0.0006). Nevertheless, KRT12 protein level did not differ between any of the groups (*p* ≥ 0.098) (Fig 3A).

Moreover, upon IL-1β induced inflammation, *CRABP2* expression was significantly upregulated in both FABP5 knockdown and control siRNA transfected cells (*p* = 0.001 and *p* = 0.017, respectively). Under treatment with IL-1β, FABP5 knockdown cells expressed more *CRABP2* mRNA than ctrl siRNA LECs (*p* = 0.034). No significant alteration was detected in *ABCG2* expression levels (*p* ≥ 0.202) (Fig 3A). There was no detectable CRABP2 protein in any of our analyzed groups, but the CRABP5 protein band was clearly visible in MCF cells, which are known to express CRABP5 at significant levels and were therefore chosen as the positive control (S1 Fig).

### FABP5 regulates gene expression by controlling PAX6 and FOSL2 expression during inflammation

**Fig 4:**
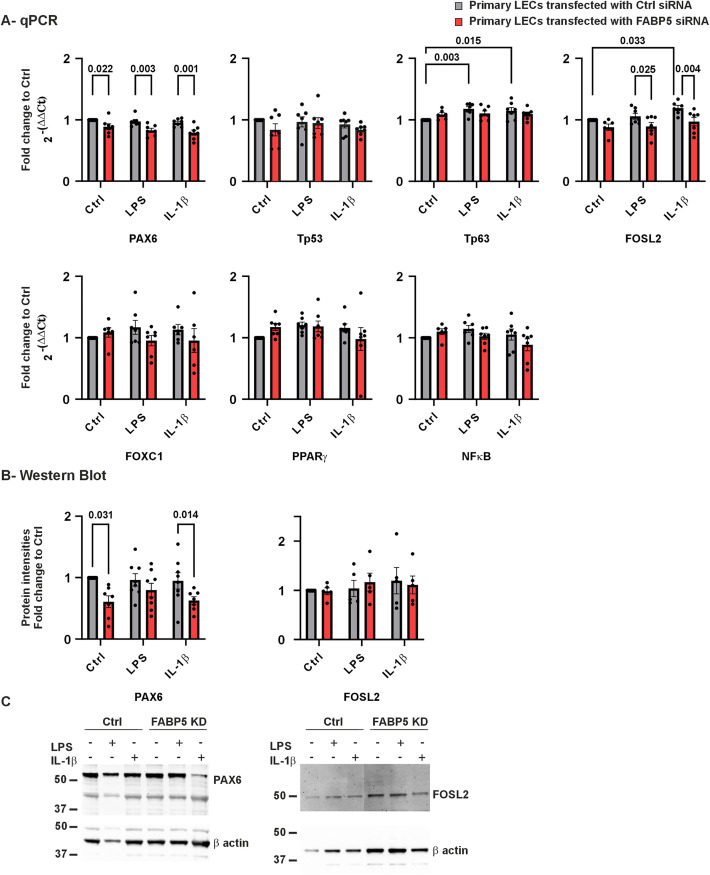
PAX6, TP53, TP63, FOSL2, FOXC1, PPARγ, NFκB mRNA and PAX6/FOSL2 proteins in LECs after FABP5 siRNA ± LPS/IL-1β. PAX6, TP53, TP63, FOSL2, FOXC1, PPARγ and NFκB mRNA expression (A) and PAX6 and FOSL2 protein levels (B) in LECs transfected with FABP5 siRNA or control (ctrl) siRNA. Western blot image from a single biological replicate **(C)**. Data are shown as mean ± SEM. Statistical analysis has been performed using two-way ANOVA followed by Tukey test (n = 7). p-values below 0.05 were considered statistically significant. PAX6 mRNA level was significantly lower in FABP5 knockdown LECs than in ctrl siRNA LECs, in KSFM medium or following LPS and IL-1β treatment (p ≤ 0.022). Furthermore, PAX6 protein level was significantly lower following FABP5 knockdown, than in ctrl siRNA LECs without treatment and after IL-1β treatment (p ≤ 0.031). TP63 mRNA level was significantly higher in ctrl siRNA LECs following LPS or IL-1β treatment, than without treatment (p ≤ 0.015). FOSL2 mRNA level was significantly higher in ctrl siRNA LECs following IL-1β treatment, than without treatment (p = 0.033). Under LPS and IL-1β treatment, FOSL2 mRNA levels were significantly lower in FABP5 knockdown cells than in control (transfected) cells (p ≤ 0.025).

Next, the effects of FABP5 deficiency on the expression levels of several key transcription factors in human primary LECs were analyzed. Among these, only the *PAX6* mRNA level was significantly decreased under all three conditions of FABP5 deficiency (*p* ≤ 0.022). The *FOSL2* mRNA level remained unchanged in KSFM medium; however, significant downregulation was observed under inflammatory conditions induced by either LPS or IL-1β (*p* ≤ 0.033). A significant increase in *TP63* (tumor protein p53) mRNA expression was observed in ctrl siRNA LECs cells upon inflammation induced by either LPS or IL-1β (*p* ≤ 0.015). The mRNA expression levels of *TP53* (tumor protein p53), *TP63*, *FOXC1* (Fork head box C1 protein), *PPARγ* (Peroxisome proliferator-activated receptors) and *NFκB* (nuclear factor ‘kappa-light-chain-enhancer’ of activated B-cells) were not significantly altered by FABP5 knockdown under any of the mentioned conditions (*p* ≥ 0.106) (Fig 4A). Intracellular protein levels of the altered genes were analyzed by western blot (Fig 4B, 4C). PAX6 protein level was significantly downregulated in FABP5 knockdown cells, compared to ctrl siRNA LECs, using KSFM medium (*p* = 0.031) and upon IL-1β induced inflammation (*p* = 0.014). FOSL2 protein levels did not differ significantly between the analyzed groups (*p* ≥ 0.6).

### IL-6 and PTGES2 mRNA expression is altered upon FABP5 downregulation

**Fig 5:**
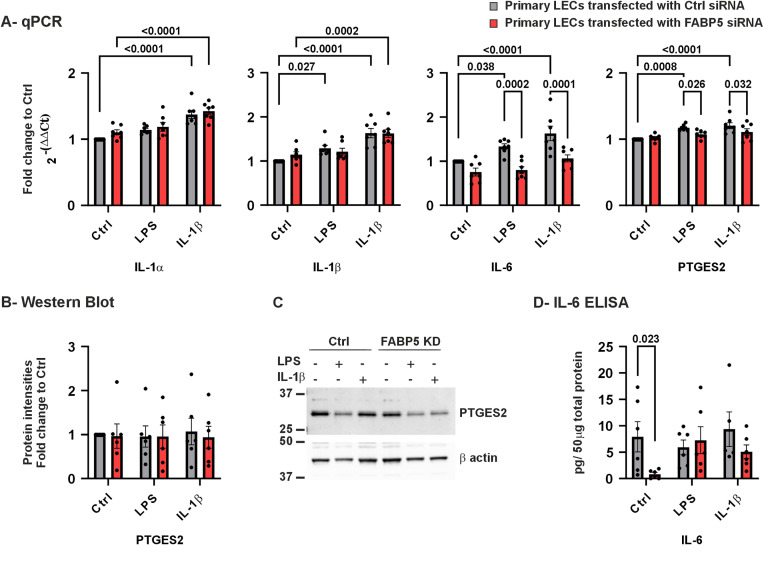
*IL-1α, IL-1β, IL-6, PTGES2* mRNA, PTGES2 protein, and lysate IL-6 in LECs after FABP5 siRNA ± LPS/IL-1β. *IL-1α, IL-1β, IL-6*, and *PTGES2* mRNA expression (A) and PTSG1 protein levels (B) in LECs transfected with FABP5 siRNA or control (ctrl) siRNA. Western blot image from a single biological replicate **(C)**. IL-6 protein levels have been determined in total protein extracted from cells, using ELISA **(D)** Data are shown as mean ± SEM. Statistical analysis has been performed using two-way ANOVA followed by Tukey test (*n = 7*). *p-*values below 0.05 were considered statistically significant. *IL-1α, IL-1β, IL-6* and *PTGES2* mRNA level were significantly higher in ctrl siRNA LECs following IL-1β treatment, than without treatment (*p* < 0.0001 for all). *IL-1α* and *IL-1β* mRNA levels were also significantly higher in FABP5 knockdown LECs after IL-1β treatment, than without treatment (*p* ≤ 0.0002). Furthermore, *IL-1β, IL-6* and *PTGES2* mRNA level were significantly higher in ctrl siRNA LECs following LPS treatment, than without treatment (*p* ≤ 0.038 for all). In FABP5 knockdown LECs, *IL-6* and *PTGES2* mRNA levels were significantly lower, than in ctrl siRNA LECs after LPS or IL-1β treatment (*p* ≤ 0.032). IL-6 protein levels in lysed cell pellets was significantly lower in FABP5 knockdown LECs without treatment, than in ctrl siRNA LECs without treatment (*p* = 0.023).

The role of FABP5 in controlling inflammation has been well studied. Therefore, we determined mRNA expression levels of the pro-inflammatory cytokines *IL-1α*, *IL-1β*, *IL-6* and *PTGES2* (Fig 5A-5D). In ctrl siRNA LECs, *IL-1α* mRNA levels were significantly increased upon stimulation by IL-1β (*p* < 0.0001). Additionally, the expression of *IL-1β*, *IL-6* and *PTGES2* was significantly upregulated in response to inflammation induced by both stimuli (*p* ≤ 0.038). On the other hand, in FABP5 deficient LECs, only treating the cells with IL-1β, led to a significant upregulation of *IL-1α* and *IL-1β* (*p* < 0.0001). Comparing control and FABP5 siRNA transfected cells revealed significantly lower mRNA expression levels of *IL-6* and *PTGES2* in FABP5 knockdown cells under both inflammatory conditions (*p* ≤ 0.032).

Western blot analysis of PTGES2 protein level showed no significant difference between the analyzed groups (*p* ≥ 0.805) (Fig 5B, 5C). Intracellular IL-6 protein concentrations were measured by ELISA, using proteins extracted from cell lysates. In KSFM medium, a significantly lower IL-6 protein level was observed in siRNA knockdown LECs compared to ctrl siRNA LECs (*p* = 0.023). In addition, IL-6 protein level tended to be significantly lower in siRNA knockdown LECs, than in ctrl siRNA LECs, using IL-1β stimulation (*p* = 0.179) (Fig 5D).

## Discussion

In the present study, we investigated the role of FABP5 in regulating gene expression programs controlling LECs proliferation, differentiation, migration, and inflammatory signaling. Given that FABP5 downregulation has previously been reported in congenital aniridia in the context of PAX6 haploinsufficiency [18], we hypothesized that reduced FABP5 expression may represent a secondary but functionally relevant event contributing to progressive epithelial dysfunction in AAK. Our results demonstrate that FABP5 deficiency significantly alters the expression of genes central to epithelial homeostasis, including *PAX6*, *KRT3*, and *MMP2*, at both mRNA and protein levels, independent of inflammatory stimulation. Under inflammatory conditions induced by LPS or IL-1β, additional transcriptional changes were observed in *FOSL2*, *VEGF-A*, *MAPK3*, *MMP2, CRABP2*, *IL-6*, and *PTGES2*, suggesting that FABP5 plays a particularly important modulatory role in stressed epithelial environments.

### FABP5 and PAX6-dependent transcriptional regulation

A central finding of this study is the observed association between FABP5 deficiency and reduced *PAX6* expression at both mRNA and protein levels (Fig 4). While *PAX6* has previously been shown to regulate *FABP5* expression [18,29], our data suggest that reduced *FABP5* levels are accompanied by further decreases in *PAX6* mRNA and protein, particularly under inflammatory conditions. We hypothesize that in patients with congenital aniridia, reduced FABP5 expression due to PAX6 haploinsufficiency, in combination with an inflammatory environment, results in a further reduction of PAX6 gene and protein expression, which can lead to increased severity of AAK. Although the effect of PAX6 on FABP5 expression has been reported in several studies [18,29], our results suggest a novel role for FABP5 in the regulation of PAX6 gene and protein expression, indicating a regulatory feedback loop.

In congenital aniridia, where *PAX6* haploinsufficiency already compromises baseline expression, inflammation-associated *FABP5* downregulation may exacerbate *PAX6* suppression, potentially reinforcing epithelial instability. Given the central role of *PAX6* in maintaining corneal epithelial homeostasis and differentiation, such a feedback mechanism could represent a critical contributor to the progression and severity of AAK.

Consistent with this concept, *FOSL2*, a transcription factor involved in epithelial proliferation and differentiation [30,31], was downregulated under inflammatory conditions in FABP5-deficient cells. A recent study by Smits et al. [31] identified a novel role for FOSL2 in maintaining limbal epithelial homeostasis and corneal transparency. In the referenced study, FOSL2 deficiency in LECs led to significant upregulation of genes associated with “negative regulation of cell proliferation” and “endothelial cell differentiation” [31], underscoring its role in maintaining epithelial homeostasis and corneal transparency. Accordingly, reduced *FOSL2* expression in FABP5 knockdown LECs may contribute to diminished proliferative capacity. Consistent with this, BrdU assays revealed reduced proliferation under LPS stimulation, whereas short-term Ki67 expression remained unchanged, suggesting that longer-term observation may be required to fully capture proliferative consequences.

### Effects on epithelial differentiation markers: KRT3 and KRT12

FABP5 deficiency was consistently associated with reduced *KRT3* expression at both mRNA and protein levels. As *KRT3* is a well-established differentiation marker of corneal epithelial cells and has been reported to be downregulated in congenital aniridia [32], these findings suggest that FABP5 contributes to maintaining epithelial differentiation status.

*KRT12*, which forms intermediate filaments together with KRT3 [33], showed context-dependent regulation. FABP5 knockdown cells exhibited increased *KRT12* mRNA expression under non-inflammatory conditions, whereas IL-1β stimulation led to a decrease in *KRT12* mRNA levels in FABP5-deficient cells. (**Fig 3**). Given that *PAX6* has been shown to regulate the *KRT12* promoter [34], these alterations may reflect complex interactions between *FABP5*, *PAX6*, and inflammatory signaling. Together, the dysregulation of *KRT3* and *KRT12* indicates structural and differentiation imbalance in FABP5-deficient LECs.

### Inflammatory modulation: IL-6, VEGF-A, PTGES2, and MAPK3

FABP5 deficiency significantly altered inflammatory signaling responses. Notably, *IL-6* mRNA upregulation in response to LPS or IL-1β was attenuated in FABP5 knockdown cells, and intracellular IL-6 protein levels remained reduced (Fig 5). Our qPCR findings are in agreement with a previous study of Babaev et al. using mouse peritoneal macrophages, where LPS induced lower *IL-6* mRNA expression in macrophages from FABP5 double knockout mouse, than in controls [35]. Similar observations have been reported in another FABP5-deficient macrophage model [15], suggesting a conserved role for FABP5 in inflammatory regulation.

Although *IL-6* is classically considered pro-inflammatory, it also promotes corneal epithelial migration and wound healing [36,37]. Therefore, reduced IL-6 expression in FABP5-deficient LECs may impair regenerative responses over time.

In addition, *VEGF-A* transcription was significantly increased in FABP5 knockdown cells under inflammatory conditions (Fig 2). Given the established role of VEGF-A in corneal neovascularization in AAK [38,39]4/8/24 9:52:00 AM, this finding suggests that FABP5 may influence the VEGF-VEGFR axis in the diseased cornea.

*PTGES2*, a mediator of prostaglandin synthesis and angiogenic signaling [40]4/8/24 9:52:00 AM, was reduced at the mRNA level under inflammatory conditions, although protein levels remained unchanged (Fig 5).

Activation of MAPK3 has been shown to be crucial for rapid initiation of wound healing in LECs [41]. An upregulation of MAPK3 has been observed in *PAX6*^*+/-*^ mouse corneal epithelial cells in resting conditions, without wounding [42]. Enhanced MAPK3 signaling has also been reported in aniridia conjunctival cells [17]. Similar to *PTGES2*, *MAPK3* transcription was decreased in LPS-treated FABP5-deficient cells, without corresponding protein alterations. These discrepancies likely reflect post-transcriptional regulation or differences in protein turnover kinetics but nonetheless indicate transcriptional reprogramming upon FABP5 loss.

### Extracellular matrix remodeling and retinoic acid signaling

FABP5 deficiency also affected genes involved in extracellular matrix remodeling. In our present study, reduced *MMP2* mRNA expression was detected in FABP5-deficient cells both in the absence of inflammation and under LPS-induced inflammatory conditions. Nevertheless, a reduction in MMP2 protein levels was observed only in cells cultured in KSFM medium upon FABP5 knockdown (Fig 2). As MMP2 contributes to basement membrane and extracellular matrix remodeling [11,43], its downregulation may alter tissue remodeling capacity.

Furthermore, inflammatory stimulation increased *CRABP2* expression while FABP5 levels were reduced, leading to an elevated CRABP2/FABP5 ratio. The CRABP2/FABP5 ratio plays a key role in regulating cellular responses to retinoic acid. An increased ratio shifts retinoic acid signaling toward growth inhibition, whereas a decreased ratio favors cell proliferation and survival [44]. In the present study, an increased *CRABP2* expression was detected under IL-1β-stimulated inflammatory conditions both in FABP5 knockdown and ctrl siRNA LECs. Therefore, CRABP2 expression was increased, whereas FABP5 expression was decreased, resulting in an elevated CRABP2/FABP5 ratio, which may, in the long term, contribute to cell growth inhibition. In congenital aniridia, reduced FABP5 expression in the context of a chronic inflammatory microenvironment may promote upregulation of CRABP2, which in turn could impair limbal epithelial cell proliferation and compromise epithelial maintenance.

## Conclusions

**Fig 6:**
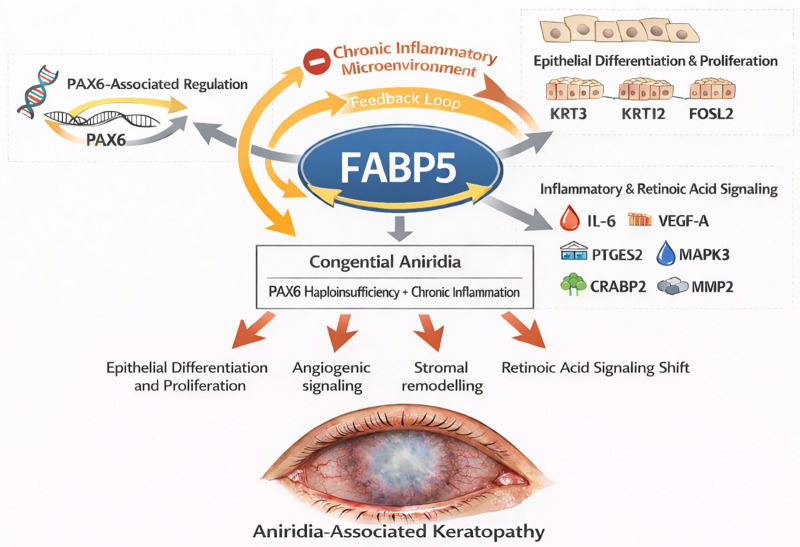
Summary of observations upon FABP5 downregulation and its possible effects on LECs physiology. Schematic representation of FABP5 as a central regulatory hub integrating three major functional axes in limbal epithelial cells: (1) PAX6-associated transcriptional regulation; (2) epithelial differentiation and proliferation control involving KRT3, KRT12, and FOSL2; and (3) inflammatory and retinoic acid signaling pathways, including IL-6, VEGF-A, PTGES2, MAPK3, CRABP2, and MMP2.

In summary, our findings support a model in which FABP5 integrates three major regulatory axes in limbal epithelial cells (Fig 6):

1. *PAX6*-associated transcriptional regulation
2. Epithelial differentiation markers (*KRT3*, *KRT12*) and proliferation-related factors (*FOSL2*)
3. Inflammatory and retinoic acid signaling pathways (*IL-6*, *VEGF-A*, *PTGES2*, *MAPK3*, *CRABP2*, *MMP2*)

In congenital aniridia, where *PAX6* haploinsufficiency and chronic inflammation coexist, reduced FABP5 expression may amplify epithelial instability, promote angiogenic signaling, impair wound healing responses, and shift retinoic acid signaling toward growth inhibition. Collectively, these alterations may contribute to AAK progression.

Future mechanistic studies, including rescue experiments restoring FABP5 expression and in vivo models, will be required to validate these interactions and determine whether FABP5 modulation represents a therapeutic strategy for stabilizing epithelial homeostasis in congenital aniridia.

In congenital aniridia, characterized by PAX6 haploinsufficiency and chronic inflammation, reduced FABP5 expression may modify epithelial differentiation and proliferation, angiogenic signaling, stromal remodelling and shift retinoic acid signaling toward growth inhibition. Collectively, these interconnected alterations may contribute to the progression of aniridia-associated keratopathy (AAK). This model highlights FABP5 as a potential therapeutic target; however, further mechanistic studies, including rescue experiments and in vivo validation, are required to confirm these proposed interactions.

## Limitations

Several limitations of this study should be acknowledged. First, the temporal design may have influenced the observed protein expression patterns. Inflammatory stimulation was performed for 24 hours, and cells were harvested 72 hours after transfection. Given that protein turnover rates vary considerably, this timeframe may have been insufficient to detect changes in proteins with longer half-lives, potentially contributing to discrepancies between mRNA and protein levels.

Second, the proposed FABP5-PAX6 interaction was not mechanistically validated. Rescue experiments assessing whether restoration of FABP5 expression can normalize PAX6 levels were beyond the scope of the present study. In addition, the underlying molecular mechanisms potentially linking FABP5 and PAX6, such as PPAR-dependent signaling or lipid-mediated transcriptional regulation, were not investigated. Accordingly, the suggested feedback relationship should be interpreted as hypothetical and requires further experimental confirmation.

Third, the limited amount of available protein from the original experimental samples restricted cytokine analysis to IL-6. Although IL-6 is a well-established marker of inflammatory activity, a broader cytokine panel would have allowed for a more comprehensive characterization of the inflammatory milieu.

Furthermore, discrepancies between mRNA and protein expression levels were observed for several targets, including KRT12, VEGFα, MAPK3 and PTGES2, and where transcriptional changes were not consistently paralleled by corresponding alterations at the protein level. Such divergence is not unexpected and may reflect complex post-transcriptional regulatory mechanisms, including microRNA-mediated control, differences in mRNA translation efficiency, variable protein turnover rates, or altered protein stability. In addition, temporal dynamics may contribute, as mRNA expression changes can precede detectable protein alterations, and single time-point analyses may therefore fail to capture these shifts. Technical factors, such as differences in assay sensitivity or detection thresholds, may also play a role. These discrepancies underscore the importance of cautious interpretation of transcriptomic data and highlight the need for integrated multi-level analyses in future studies to more comprehensively characterize molecular alterations.

Future studies incorporating longer observation periods and in vivo models are warranted to more thoroughly evaluate the effects of FABP5 modulation on limbal epithelial cell function and to validate the proposed molecular pathways.

## Supporting information

S1 Fig Original, uncropped, and unadjusted images underlying all Western blot results are presented (A-I). (PDF)

S1 Table Relative mRNA expression in control or FABP5 siRNA LECs ± LPS/IL-1β (mean ± SEM). Quantitative PCR (qPCR) analysis was performed to determine relative mRNA expression levels of the indicated genes in LECs. All values were normalized within each experiment to control siRNA LECs cultured in Ctrl medium, which were set to 1.
(DOCX)

S2 Table Relative protein expression in control or FABP5 siRNA LECs ± LPS/IL-1β (mean ± SEM). Values are normalized to control siRNA LECs cultured in Ctrl medium, which were set to 1. FABP5, VEGF-A, ERK1/2, MMP2, KRT3, KRT12, PAX6, FOSL2, and PTGES2 protein levels were measured.
(DOCX)
