# Supplementary material for: Fatty acid-binding protein 5 (FABP5) modulates limbal epithelial cell homeostasis by regulating the expression of key genes under both normal and inflammatory conditions, in vitro
Source: PLoS One. 2026 Apr 28;21(4):e0347228. doi: 10.1371/journal.pone.0347228 (PMC13123934; doi:10.1371/journal.pone.0347228)
Supplement: S1 Fig — (PDF) [file pone.0347228.s001.pdf]

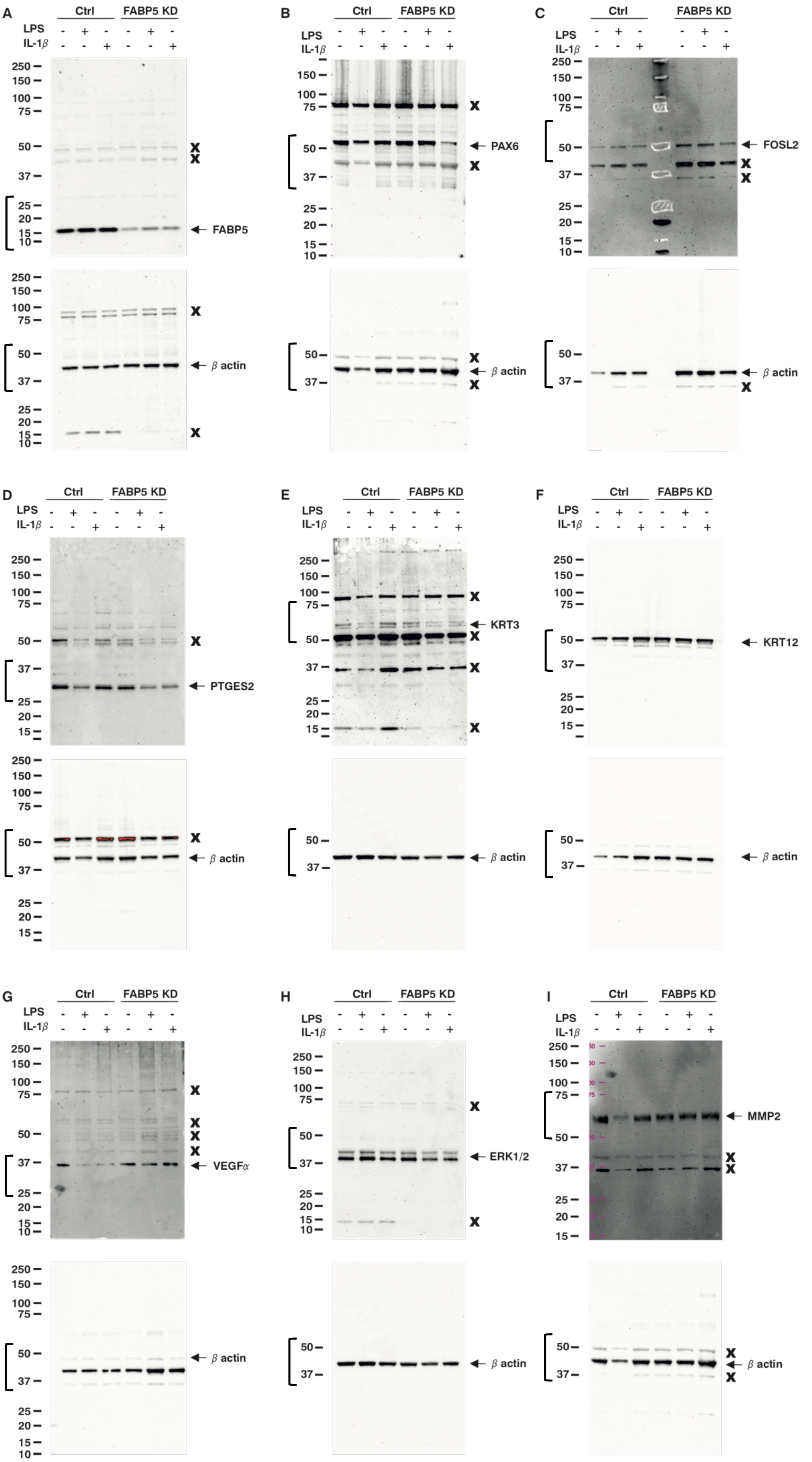

S1 Fig. Original, uncropped, and unadjusted images underlying all Western blot results and their corresponding β-actin lane are presented (A-I). Lanes which are not included in the final figure are marked with "X" and "[" indicates the frame used in the manuscript.
