# Supplementary material for: Fatty acid-binding protein 5 (FABP5) modulates limbal epithelial cell homeostasis by regulating the expression of key genes under both normal and inflammatory conditions, in vitro
Source: PLoS One. 2026 Apr 28;21(4):e0347228. doi: 10.1371/journal.pone.0347228 (PMC13123934; doi:10.1371/journal.pone.0347228)
Supplement: S1 Table — Quantitative PCR (qPCR) analysis was performed to determine relative mRNA expression levels of the indicated genes in LECs. All values were normalized within each experiment to control siRNA LECs cultured in Ctrl medium, which were set to 1. (DOCX) [file pone.0347228.s002.docx]

**S1 Table. Relative mRNA expression in control or FABP5 siRNA LECs ± LPS/IL-1β (mean ± SEM).** Quantitative PCR (qPCR) analysis was performed to determine relative mRNA expression levels of the indicated genes in LECs. All values were normalized within each experiment to control siRNA LECs cells cultured in Ctrl medium, which were set to 1(mean ± SEM).

| **qPCR data** |  | Control siRNA | | FABP5 siRNA | |
| --- | --- | --- | --- | --- | --- |
| Gene name | Medium conditions | Mean | SEM | Mean | SEM |
| *FABP5* | Ctrl | 1 | 0 | 0.0736434 | 0.0089046 |
|  | LPS | 1.045525 | 0.0374273 | 0.0696434 | 0.0073665 |
|  | IL-1*β* | 1.1426843 | 0.029131 | 0.0832989 | 0.0094032 |
| *VEGFα* | Ctrl | 1 | 0 | 1.1504479 | 0.0792616 |
|  | LPS | 1.0616578 | 0.0457946 | 1.2866244 | 0.0655673 |
|  | IL-1*β* | 1.1466771 | 0.0715509 | 1.4017655 | 0.1092566 |
| *MAPK1* | Ctrl | 1 | 0 | 1.0715855 | 0.0319522 |
|  | LPS | 1.1149692 | 0.0396556 | 1.0228702 | 0.0495208 |
|  | IL-1*β* | 1.0812897 | 0.0417318 | 1.0017927 | 0.0489145 |
| *MAPK3* | Ctrl | 1 | 0 | 0.8250356 | 0.1003839 |
|  | LPS | 1.1526043 | 0.0454756 | 0.9050181 | 0.0559446 |
|  | IL-1*β* | 0.9426383 | 0.0490772 | 0.8052023 | 0.0936211 |
| *AKT1* | Ctrl | 1 | 0 | 0.9971193 | 0.0651469 |
|  | LPS | 1.1587671 | 0.1031045 | 1.0302911 | 0.0405905 |
|  | IL-1*β* | 1.0558216 | 0.0643912 | 1.0653842 | 0.0914781 |
| *MMP2* | Ctrl | 1 | 0 | 0.7251061 | 0.1049197 |
|  | LPS | 1.0129289 | 0.0597414 | 0.7733168 | 0.0544296 |
|  | IL-1*β* | 1.0317748 | 0.1206882 | 0.7931267 | 0.1055157 |
| *MMP9* | Ctrl | 1 | 0 | 1.3374838 | 0.1273511 |
|  | LPS | 1.4595052 | 0.1984549 | 1.4551324 | 0.1968335 |
|  | IL-1*β* | 1.0305725 | 0.042639 | 0.9740818 | 0.1053021 |
| *Ki67* | Ctrl | 1 | 0 | 0.937125 | 0.0662219 |
|  | LPS | 1.088 | 0.022144 | 1.067375 | 0.0570169 |
|  | IL-1*β* | 0.99875 | 0.0346811 | 0.96575 | 0.0583107 |
| *KRT3* | Ctrl | 1 | 0 | 0.7684116 | 0.0662312 |
|  | LPS | 1.1266003 | 0.0617948 | 0.7597421 | 0.040116 |
|  | IL-1*β* | 1.0883158 | 0.0236637 | 0.7655927 | 0.0435795 |
| *KRT12* | Ctrl | 1 | 0 | 1.2358283 | 0.0732489 |
|  | LPS | 1.0726647 | 0.0317128 | 1.1447513 | 0.0577093 |
|  | IL-1*β* | 1.0627774 | 0.0433286 | 0.9590189 | 0.0431814 |
| *CRABP2* | Ctrl | 1 | 0 | 1.2543956 | 0.0945424 |
|  | LPS | 1.2046863 | 0.1272723 | 1.491687 | 0.1382231 |
|  | IL-1*β* | 1.5974523 | 0.0898229 | 2.0540486 | 0.2774903 |
| *ABCG2* | Ctrl | 1 | 0 | 1.1226514 | 0.1224881 |
|  | LPS | 1.090564 | 0.0507152 | 1.0138987 | 0.0819587 |
|  | IL-1*β* | 0.9831667 | 0.0411163 | 1.121411 | 0.0781774 |
| *PAX6* | Ctrl | 1 | 0 | 0.8883543 | 0.0459978 |
|  | LPS | 0.9744534 | 0.0301494 | 0.8299305 | 0.0335891 |
|  | IL-1*β* | 0.9546269 | 0.0231309 | 0.7915372 | 0.0429862 |
| *TP53* | Ctrl | 1 | 0 | 0.8384534 | 0.0981791 |
|  | LPS | 0.9674872 | 0.0818565 | 0.9488696 | 0.088517 |
|  | IL-1*β* | 0.9259423 | 0.056878 | 0.8377915 | 0.0349736 |
| *TP63* | Ctrl | 1 | 0 | 1.0854884 | 0.0320131 |
|  | LPS | 1.1793594 | 0.0349749 | 1.1050609 | 0.0401433 |
|  | IL-1*β* | 1.1482012 | 0.0524926 | 1.0937637 | 0.0329676 |
| *FOSL2* | Ctrl | 1 | 0 | 0.8805297 | 0.0504449 |
|  | LPS | 1.0584225 | 0.0487641 | 0.8939362 | 0.0618706 |
|  | IL-1*β* | 1.1921187 | 0.0434068 | 0.9710064 | 0.0682933 |
| *FOXC1* | Ctrl | 1 | 0 | 1.0869899 | 0.0774499 |
|  | LPS | 1.1702965 | 0.1132866 | 0.9545853 | 0.0841638 |
|  | IL-1*β* | 1.1332011 | 0.08501 | 0.9551202 | 0.1945002 |
| *PPARγ* | Ctrl | 1 | 0 | 1.1737697 | 0.0573451 |
|  | LPS | 1.2063601 | 0.047997 | 1.1856549 | 0.0873177 |
|  | IL-1*β* | 1.1601294 | 0.0663579 | 0.9801875 | 0.1869198 |
| *NFκB* | Ctrl | 1 | 0 | 1.0997328 | 0.0474497 |
|  | LPS | 1.1445516 | 0.0562763 | 1.0246378 | 0.0507326 |
|  | IL-1*β* | 1.0533404 | 0.0895784 | 0.8865377 | 0.0970476 |
| *IL-1α* | Ctrl | 1 | 0 | 1.1040025 | 0.0381782 |
|  | LPS | 1.1416636 | 0.022649 | 1.1879122 | 0.0654743 |
|  | IL-1*β* | 1.3740328 | 0.0610283 | 1.4241476 | 0.0488548 |
| *IL-1β* | Ctrl | 1 | 0 | 1.1456236 | 0.0648878 |
|  | LPS | 1.2885732 | 0.0680061 | 1.2135297 | 0.0765063 |
|  | IL-1*β* | 1.6317009 | 0.1061427 | 1.6269243 | 0.0898934 |
| *IL-6* | Ctrl | 1 | 0 | 0.7537028 | 0.0893708 |
|  | LPS | 1.3328574 | 0.0609625 | 0.8007197 | 0.0761087 |
|  | IL-1*β* | 1.6278171 | 0.1645844 | 1.0620345 | 0.0789843 |
| *PTGES2* | Ctrl | 1 | 0 | 1.022522 | 0.0189916 |
|  | LPS | 1.1723752 | 0.0175317 | 1.0731594 | 0.0226391 |
|  | IL-1*β* | 1.2042486 | 0.0439862 | 1.1093028 | 0.0487048 |
