# Supplementary material for: Fatty acid-binding protein 5 (FABP5) modulates limbal epithelial cell homeostasis by regulating the expression of key genes under both normal and inflammatory conditions, in vitro
Source: PLoS One. 2026 Apr 28;21(4):e0347228. doi: 10.1371/journal.pone.0347228 (PMC13123934; doi:10.1371/journal.pone.0347228)
Supplement: S2 Table — Values are normalized to control siRNA LECs cultured in Ctrl medium, which were set to 1. FABP5, VEGF-A, ERK1/2, MMP2, KRT3, KRT12, PAX6, FOSL2, and PTGES2 protein levels were measured. (DOCX) [file pone.0347228.s003.docx]

**S2 Table. Relative protein expression in control or FABP5 siRNA LECs ± LPS/IL-1β (mean ± SEM).** Values are normalized to control siRNA LECs cultured in Ctrl medium, which were set to 1. FABP5, VEGF-A, ERK1/2, MMP2, KRT3, KRT12, PAX6, FOSL2, and PTGES2 protein levels were measured (mean ± SEM).

|  |  | Control siRNA | | FABP5 siRNA | |
| --- | --- | --- | --- | --- | --- |
| Protein | Medium conditions | Mean | SEM | Mean | SEM |
| FABP5 | Ctrl | 1 | 0 | 0.2342479 | 0.0343489 |
|  | LPS | 0.8811068 | 0.0829195 | 0.2990447 | 0.0724222 |
|  | IL-1β | 1.1391393 | 0.1913783 | 0.3238157 | 0.0782714 |
| VEGFα | Ctrl | 1 | 0 | 0.9319702 | 0.3316745 |
|  | LPS | 0.8619304 | 0.3441703 | 0.8629502 | 0.3518579 |
|  | IL-1β | 1.652073 | 0.5700777 | 1.1684937 | 0.3661741 |
| ERK1/2 | Ctrl | 1 | 0 | 1.096251 | 0.14977 |
|  | LPS | 1.365965 | 0.0299282 | 1.1716267 | 0.1709139 |
|  | IL-1β | 1.2473598 | 0.1806441 | 1.1175004 | 0.1517456 |
| MMP2 | Ctrl | 1 | 0 | 0.4246348 | 0.1214723 |
|  | LPS | 0.5226143 | 0.1073425 | 0.3963872 | 0.1060829 |
|  | IL-1β | 0.5582624 | 0.0753714 | 0.3668087 | 0.0981291 |
| KRT3 | Ctrl | 1 | 0 | 0.5720208 | 0.0736922 |
|  | LPS | 0.9692809 | 0.1873598 | 0.7910225 | 0.1916828 |
|  | IL-1β | 1.0636568 | 0.1146304 | 0.6241755 | 0.0885726 |
| KRT12 | Ctrl | 1 | 0 | 0.6943838 | 0.1356616 |
|  | LPS | 0.8419205 | 0.1129286 | 0.8179645 | 0.166188 |
|  | IL-1β | 0.7420691 | 0.1766832 | 0.6032467 | 0.0801337 |
| PAX6 | Ctrl | 1 | 0 | 0.6075478 | 0.0963907 |
|  | LPS | 0.9634499 | 0.1010238 | 0.7973883 | 0.1113339 |
|  | IL-1β | 0.09488673 | 0.1337457 | 0.6272337 | 0.0623203 |
| FOSL2 | Ctrl | 1 | 0 | 0.9808086 | 0.0693914 |
|  | LPS | 1.0404689 | 0.1633986 | 1.1662693 | 0.182351 |
|  | IL-1β | 1.1954161 | 0.2674974 | 1.1100308 | 0.1813467 |
| PTGES2 | Ctrl | 1 | 0 | 0.9649454 | 0.2766238 |
|  | LPS | 0.9550972 | 0.2419701 | 0.9535777 | 0.266555 |
|  | IL-1β | 1.0688727 | 0.3008595 | 0.9375076 | 0.2492872 |
